# Current expectations of the arterial switch operation in a small volume center: a 20-year, single-center experience

**DOI:** 10.1186/s13019-016-0428-9

**Published:** 2016-02-24

**Authors:** Man-shik Shim, Tae-Gook Jun, Ji-Hyuk Yang, Pyo Won Park, I Seok Kang, June Huh, Jin Young Song

**Affiliations:** Department of Thoracic and Cardiovascular Surgery, Samsung Medical Center, Sungkyunkwan University School of Medicine, 50 Irwon-dong, Kangnam-gu, Seoul, 135-710 Korea; Department of Pediatrics, Samsung Medical Center, Sungkyunkwan University School of Medicine, Seoul, Korea

**Keywords:** Congenital heart defect, Arterial switch operation, Transposition of the great arteries, Double outlet right ventricle with subpulmonary ventricular septal defect, Taussig-Bing anomaly

## Abstract

**Background:**

We reviewed our 20-year experience with arterial switch operation (ASO) for transposition of the great arteries (TGA) or double outlet right ventricle with subpulmonary ventricular septal defect (Taussig-Bing anomaly) to assess the early and long-term outcomes.

**Methods:**

Between January 1995 and December 2014, 139 consecutive patients who underwent ASO for TGA or Taussig-Bing anomaly were included in this retrospective study. The median age at the operation was 9 (0–485) days, and 97 patients (70 %) underwent ASO less than 2 weeks. The median weight was 3.3 (2.1-10.3) kg. The patients were divided into three groups; simple TGA (*n* = 78) included patients with TGA with intact ventricular septum, complex TGA (*n* = 46) included those who had TGA with ventricular septal defect or other anomalies, and Taussig-Bing anomaly (*n* = 15). Median follow-up duration was 72.5 (0.4-230) months.

**Results:**

There were 3(2.2 %) in-hospital deaths. One patient (0.7 %) underwent early reoperation due to coronary insufficiency. Late deaths occurred in 3 (2.2 %) of 136 survivors. The Kaplan-Meier’s survival rate was 97.6 ± 1.4 % at 15 years. Twenty-three patients (16.9 %) required 26 reintervention. The freedom from reintervention rates were 82.5 ± 3.7 % at 5 years and 75.8 ± 4.7 % at 10 years, respectively. Median interval between ASO and first reintervention was 22.8 (6.4-89.2) months. The multivariate analysis showed that diagnosis of Taussig-Bing anomaly (hazard ratio, 7.09; *P* < 0.001) and side by side great artery relationship (hazard ratio, 7.98; *P* = 0.001) were independent risk factors for reoperation. Five patients (3.9 %) had developed at least moderate neo-aortic regurgitation during the follow-up and one patient underwent reoperation mainly for neo-aortic regurgitation. By multivariate analysis, Taussig-Bing anomaly was the risk factor for at least moderate neo-aortic regurgitation (*P* = 0.035).

**Conclusions:**

ASO can be performed with a low risk of early mortality and satisfactory long-term outcomes even in a small volume center. Close long-term surveillance is mandatory to detect structural or hemodynamic changes.

## Background

The arterial switch operation (ASO) has become the procedure of choice for the transposition of great arteries (TGA) and double outlet right ventricle (DORV) with subpulmonary ventricular septal defect (VSD) (Taussig–Bing anomaly, TBA). Since the first successful ASO reported by Jatene et al. in 1975 [1], there have been a steadily improvements in diagnosis, surgical techniques, and perioperative management. The result of ASOs has been improved and the reported mortality has fallen to the range of 0%–6%, even when the complex group is included [2–5]. However, anatomic variations of the coronary artery, combined arch anomalies, low birth weight, and age presentation over 4 weeks are still considered risk factors [3, 4, 6].

The purpose of this study was to review the ASO at a single institution with a small volume. This report focuses on the short- and midterm results according to the anatomic subtype and surgical techniques. In addition, it intends to determine the current risk factors for mortality and morbidity.

## Methods

We conducted a retrospective review of patients who had an ASO with the diagnosis of d-TGA or DORV (TBA) from January 1995 to October 2014. A total of 139 consecutive patients were included in the study. Patients with various forms of TGA undergoing palliative ASO, double-switch operation, and half-turned switch operation were excluded from this study. Patient characteristics are shown in Table 1. The patients were divided into 3 groups according to diagnosis. The simple TGA group (*n* = 78) included patients with TGA with an intact ventricular septum. The complex TGA group (*n* = 46) included those who had d-TGA with a VSD or other anomalies such as coarctation of the aorta, interrupted aortic arch, total anomalous pulmonary venous return, significant left ventricular outflow tract (LVOT) obstruction, and those who had l-TGA with situs inversus. The TBA group (*n* = 15) included patients who were diagnosed with this anomaly. Permission to perform a retrospective review of medical records was obtained from the Institutional Review Board of Samsung Medical Center. The need for individual consent for the study was waived.

**Table 1:** Demographic data and anatomic characteristics of 139 patients who underwent an arterial switch operation

			Simple TGA	Complex TGA	Taussig-Bing Anomaly	*p* value	Total
			*n* = 78	*n* = 46	*n* = 15		*n* = 139
Age, days			7 (1–48)	12.5 (2–300)	17 (6–485)	<0.001	9 (1–485)
Age (≤14 days)			66 (84.6)	24 (52.2)	7 (46.7)	<0.001	97 (69.8)
Weight, kg			3.3 (2.4–4.8)	3.3 (2.1–7.7)	3.8 (2.8–10.3)	0.063	3.3 (2.1–10.3)
Female			13 (16.7)	17 (38.6)	6 (40.0)	0.016	36 (25.9)
Previous interventions			32 (41.0)	22 (47.8)	9 (60.0)	0.381	63 (45.3)
	BAS		32 (41.0)	20 (43.5)	8 (53.3)	0.680	60 (43.2)
	PAB, PDA ligation		0	1	0		1
	PAB, Atrial septostomy		0	0	1		1
	Modified BT shunt		0	1	0		1
Associated anomalies							
	VSD		0 (0)	43 (93.5)	15 (100)	<0.001	58 (41.7)
	Multiple VSD		0 (0)	4 (8.7)	0 (0)	0.022	4 (2.9)
	Aortic arch anomaly		1 (1.3)	5 (10.9)	10 (66.7)	<0.001	16 (11.5)
		Coarctation of aorta	0 (0)	5 (10.9)	7 (46.7)	<0.001	12 (8.6)
		Interrupted aortic arch	0 (0)	0 (0)	2 (13.3)	0.011	2 (1.4)
		Double aortic arch	0 (0)	0 (0)	1 (6.7)	0.108	1 (0.7)
		Retro-esophageal aortic arch	1 (1.3)	0 (0)	0 (0)	1.000	1 (0.7)
	IVC interruption		1 (1.3)	1 (2.2)	0 (0)	1.000	2 (1.4)
	Total anomalous pulmonary venous return		0 (0)	1 (2.2)	0 (0)	0.439	1 (0.7)
	LVOTO		0 (0)	1 (2.2)	0 (0)	0.439	1 (0.7)
	Situs inversus		0 (0)	3 (6.5)	0 (0)	0.065	3 (2.2)
Coronary pattern							
	1LCx;2R (Usual)		50 (64.1)	35 (76.1)	7 (46.7)	0.103	92 (66.2)
	1 L;2RCx		13 (16.7)	5 (10.9)	3 (20.0)	0.507	21 (15.1)
	1LR;2Cx		5 (6.4)	0 (0)	2 (13.3)	0.064	7 (5.0)
	1R;2LCx (Inverted)		2 (2.6)	2 (4.3)	0 (0)	0.765	4 (2.9)
	2LCxR (Single coronary)		8 (10.3)	4 (8.7)	3 (20.0)	0.485	15 (10.8)
	Intramural		6 (7.7)	5 (10.9)	1 (6.7)	0.823	12 (8.6)

TGA, transposition of great arteries; BAS, balloon atrial septostomy; PAB, pulmonary artery banding; PDA, patent ductus arteriosus; BT Blalock–Taussig; VSD, ventricular septal defect; IVC, inferior vena cava; TAPVR, total anomalous pulmonary venous return; LVOTO, left ventricular outflow tract obstruction; L, left anterior descending; Cx, circumflex; R, right coronary artery

### Definitions

Early death or reoperation was defined as death or reoperation occurring within 30 days of ASO or before hospital discharge. Late death or reoperation was defined as death or reoperation occurring after discharge and more than 30 days after ASO. Reoperation was defined as an operation on the heart or great vessels performed after the ASO, excluding exploration for bleeding, wound debridement, mechanical circulatory support, and pacemaker insertion. Reintervention included reoperation or catheter intervention performed after ASO.

### Follow-up

Most patients underwent regular outpatient follow-up visits at a pediatric cardiology clinic. Complete clinical follow-up data, which included an echocardiogram, were available for 129 of the 136 survivors (94.9 %). Median follow-up duration was 72.5 (range 0.4–230) months. Although ischemic symptoms are the indication for the coronary angiography, all patients are too young to express their symptoms during early period after the ASO. So, we routinely performed the coronary angiography one year after the ASO.

### Surgical techniques

After a standard median sternotomy, the thymus was removed. A large pericardial patch was harvested and fixed with a 0.625 % glutaraldehyde solution. Cardiopulmonary bypass (CPB) is instituted using bicaval cannulation as the usual pattern at temperatures between 25 °C and 32 °C. The ASO underwent at mild to moderate hypothermia (25 ~ 35 °C) while maintaining systemic perfusion. Routine selective cerebral perfusion or circulatory arrest had not been used except in patients with combined aortic arch obstruction such as interrupted aortic arch and coarctation of aorta. When the arch reconstruction was anticipated, the arterial cannula is connected to the innominate artery via 3.5-mm Gore-Tex tube interposition graft. During the arch repair, the selected antegrade cerebral perfusion was conducted at >70 ml/kg/min at the lowest rectal temperature of 25 °C.

Sharp and blunt dissection of the ascending aorta and arch vessels, pulmonary arteries, and both hilum was conducted carefully before the aortic cross-clamping. Myocardial protection was achieved by multidose antegrade cold blood cardioplegia. After dividing the ascending aorta, a cardioplegic solution was infused through the divided proximal ascending aorta or directly into the coronary artery using a small coronary catheter Before 2002, the coronary artery transfer was achieved using the common technique of performing the coronary translocation with the neoaortic root open distally. From 2002, a neoaorta was reconstructed first and then followed by a coronary artery transfer [7]. Inadequate coronary transfer causes aortic root distortion which can lead to the development of coronary stenosis, neoaortic root dilatation, and neoaortic regurgitation or stenosis. With the open technique, it is difficult to decide the adequate location of coronary transfer because of collapsed aorta. Although the early mortality rate was low with open technique, we warried about long term outcomes. By using the closed technique, the location of coronary button transfer can be determined more easily and more accurately while the aorta is inflated. Additionally, the bleeding from anastomosis site can be confirmed more conveniently and it makes the procedure easier. After cardioplegic arrest, the ascending aorta was divided at the level of 10 mm above the sinotubular junction (slightly above the level of pulmonary artery bifurcation). The main pulmonary artery was divided at the level of bifurcation. After examination of the pulmonary valve, the marking sutures for commissures were inserted at the external surface of the proximal pulmonary root. The distal pulmonary artery was dissected more distally and a Lecompte maneuver was performed. The neoaorta reconstruction was completed with 8–0 or 7–0 polypropylene suture material. The aortic clamp was removed and bleeding controlled along the suture line. For coronary artery transfer, a generous coronary artery button that included most of the corresponding sinus was harvested in the belief that it can allow a greater coronary artery length and prevent tension and torsion after coronary artery transfer [8]. During the coronary artery button preparation, we endeavored to minimize damage to the vasa vasorum of the coronary artery and to avoid transecting the proximal branches of coronary arteries. After inflation of the coronary sinus, we constructed tagging sutures on the corresponding sinus of each coronary artery button avoiding the previously inserted sutures marking the commissures. Small stab incisions were made just outside the tagging sutures after clamping of the aorta, the orifices were widened into a C-shape to construct a trapdoor while being careful not to damage the aortic valve, and the coronary artery buttons were anastomosed with 8–0 polypropylene suture material. The neopulmonary artery root was reconstructed with a pantaloon-shaped autologous pericardial patch and then the root was anastomosed to the distal main pulmonary artery. When VSD closure was needed, the VSD was closed with a bovine pericardial patch or Dacron patch before the ASO.

### Statistical analysis

Data were collected retrospectively. Continuous variables are expressed as median (and range), and categorical variables are expressed as percentages. To compare the 3 groups, a one-way analysis of variance (ANOVA) and Kruskal–Wallis test were used for normal and skewed continuous variables, respectively, and chi-square and Fisher exact tests were used for categorical variables. Factors associated with early mortality were analyzed by multiple logistic regression. Kaplan–Meier survival analyses with a log-rank test were used to analyze late survival reintervention, reoperation, neoaortic insufficiency, and event-free survival. A Cox proportional hazards model with a forward stepwise procedure was used to evaluate risk factors for late survival, reoperation, neoaortic insufficiency, and event-free survival. Variables with *p* < 0.20 in the univariate analysis constituted the starting set of covariates and variables with *p* ≥ 0.05 were excluded from each stepwise selection. Variables analyzed in the univariate analysis were age, sex, body weight, previous palliative surgery, previous balloon atrial septostomy, diagnosis with TBA, aortic arch obstruction (coarctation of aorta, interrupted aortic arch), unusual coronary artery, single coronary artery, intramural coronary artery, CPB time, aortic cross-clamp time, postoperative open sternum, and side-by-side great artery relationship. All statistical analyses were conducted using IBM SPSS Statistics for Windows (version 22; IBM SPSS, Armonk, NY, USA). A *p* < 0.05 was considered significant.

## Results and discussion

### Perioperative characteristics

The median CPB time and mean aortic cross-clamp time were 196.5 (121–501) min and 120.5 (53–300) min, respectively. The sternum was left open in 42 of 139 patients (30.2 %) and delayed sternal closure was completed at a median of 3 (2–6) days. The duration of postoperative mechanical ventilation was 4.8 (0.6–52.9) days. The median length of stay in an intensive care unit (ICU) and the median hospital stay were 8.8 (0.8–92.8) days and 15 (1–123) days, respectively.

### Early outcomes

There were 3 (2.2 %) in-hospital deaths. The first patient was diagnosed preoperatively as having TGA with an intact ventricular septum and a coronary artery with an intramural course. He died within the first 24 hours after surgery which is presumed to be caused by myocardial ischemia. The second who had TGA with large VSD underwent the ASO at 61 days old due to delayed diagnosis. She preoperatively had pulmonary hypertension and pulmonary congestion. The pulmonary hypertensive crisis was developed on the fourth postoperative day. Medical treatments including adjustment of mechanical ventilation setting, inhaled nitric oxide, additional sedatives and pulmonary vasodilators was initiated immediately and seem to be effective. However, intractable pulmonary hypertensive crisis was developed a few hours later on the fifth postoperative day and the patient died of right heart failure. These 2 cardiac deaths occurred in the earlier period before 2001. The third who had TGA with VSD died of unknown origin of septic shock. He suffered from capillary leak syndrome immediately after the ASO and symptoms (such as hypotension, generalized edema, oliguria) worsen over time in contrast to his preserved heart function. With suspecting infection as a cause, general management of sepsis including broad-spectrum antibiotics began. However, his condition had become worse and disseminated intravascular coagulation (DIC) and vegetation on right atrium were developed. Finally, he died of multi-organ failure on the 48th postoperative day. A multivariate logistic regression analysis demonstrated that low body weight (<3 kg) at surgery was the only risk factor for early hospital mortality (odds ratio, 16.7; *p* = 0.030; Nagelkerke R^2^, 0.372).

Thirty-five early complications occurred in 32 patients (23 %). Fifteen patients (10.8 %) required operation for superficial sternal wound infection and 5 patients (3.5 %) underwent diaphragmatic plication. Five patients (3.5 %) had a chylothorax that was managed with medical treatment. Three patients (2.2 %) were re-explored because of postoperative bleeding. Two patients (1.4 %) who presented with seizure were diagnosed with intracranial hemorrhage postoperatively, but this hemorrhage revolved during the follow-up without neurologic sequelae. Two patients (1.4 %) had a small bowel perforation caused by a peritoneal dialysis catheter. One patient (0.7 %) underwent early reoperation because of coronary insufficiency caused by external compression of the neopulmonary artery. The patient had an anomaly of a single coronary artery, and we transferred the coronary artery using the trapdoor technique during the ASO. However, there was a myocardial depression during the period in ICU, so we performed pulmonary artery repair on the fourth postoperative day to relieve the coronary compression. Other complications included aspiration pneumonia in 1 patient, catheter-related infection in 1, and pericardial effusion in 1. There was no atrioventricular block.

### Late outcomes

#### Late survival

**Fig. 1:**
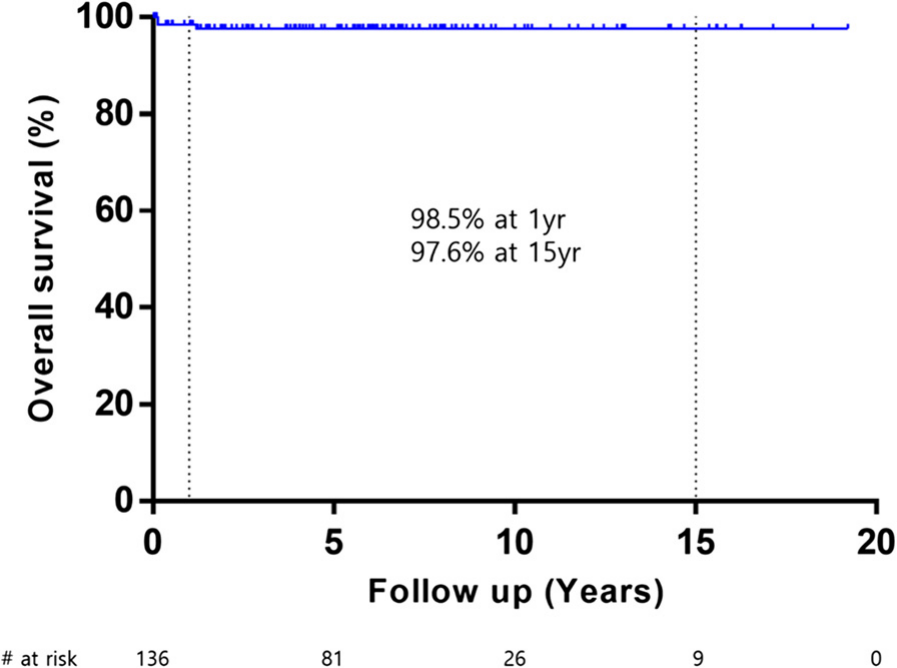
Kaplan–Meier survival curves showing overall survival after the arterial switch operation

Among the 136 patients surviving the initial operation, there were 3 (2.2 %) later deaths. Among them, the earlier two patients had TGA with an intact ventricular septum. Both patients were successfully discharged without complications on eleventh and ninth postoperative day, respectively. But they died suddenly and unexpectedly 2 months after the ASO. Unfortunately, they died outside our hospital and we could not get a detailed history. So, we could not identify their true cause of death. Nevertheless, considering the abruptness of the deaths in those patients with no problems during postoperative hospital course, we presume that the cause of death were coronary ischemia. The third patient who had TBA with coarctation of the aorta and a single coronary artery including the intramural course of the conal branch underwent ASO and coarctoplasty. This patient also underwent left subclavian artery interposition between the aorta and the conal branch because of coronary injury. After 5 months, reoperation was performed for severe aortic insufficiency. At 14 months after the initial repair, we found that severe aortic insufficiency recurred at a routine check-up and decided to reoperate electively. However, he died abruptly the day before hospital admission. Although the exact cause of death was unknown, he had symptoms of acute upper respiratory infection for a few days. Presumably, some infectious condition such as myocarditis might aggravate myocardial depression. The overall actuarial survival rates were 98.5 ± 1.1 % at 1 year and 97.6 ± 1.4 % at 15 years (Fig. 1). There were no significant risk factors for late mortality.

#### Late reintervention

During the follow-up period, 23 patients (16.9 %) subsequently required 26 reintervention including 21 reoperations in 19 patients (15.4 %) and 5 balloon angioplasties in 5 patients (3.7 %). Primary reasons for reintervention according to the groups are presented in Table 2. Multiple reinterventions were required in one patient who had TBA with an interrupted aortic arch. He underwent a first reoperation involving right ventricular outflow tract (RVOT) with a Gore-Tex monocuspid patch at 6 months after the initial surgery and underwent a second reoperation for RVOT stenosis using a monocuspid transannular homograft patch 2 years after reoperation. Seven years later, balloon angioplasty was performed for pulmonary artery stenosis and he finally underwent pulmonary valve replacement 9 years after the second surgery. Balloon angioplasty was performed for supravalvar pulmonary artery stenosis in 3 patients, for pulmonary valve stenosis in 1, and for residual coarctation of aorta in 1.

**Table 2:** Primary reasons for reintervention in 136 hospital survivors

		Simple TGA *n* = 77	Complex TGA *n* = 44	Taussig–Bing Anomaly *n* = 15	Total *n* = 136	*p*
Reintervention		2 (2.8)	0	3 (20.0)	5 (3.7)	0.008
	Supravalvar pulmonary stenosis	2 (2.6)	0	2 (13.3)	4 (2.9)	0.058
	Residual CoA	0	0	1 (6.7)	1 (0.7)	0.110
Reoperation		7 (9.1)	5 (11.4)	7 (46.7)	19 (14.0)	0.003
	Pulmonary tract lesion	4 (5.2)	3 (6.8)	5 (33.3)	12 (8.8)	0.008
	Systemic tract lesion	1 (1.3)	2 (4.5)	2 (13.3)	5 (3.7)	0.043
	Coronary artery lesion	2 (2.6)	0	0	2 (1.5)	0.631
Total		9 (11.7)	5 (11.4)	9 (60.0)	23 (16.9)	<0.001

TGA, transposition of great arteries; CoA, coarctation of aorta

**Fig. 2:**
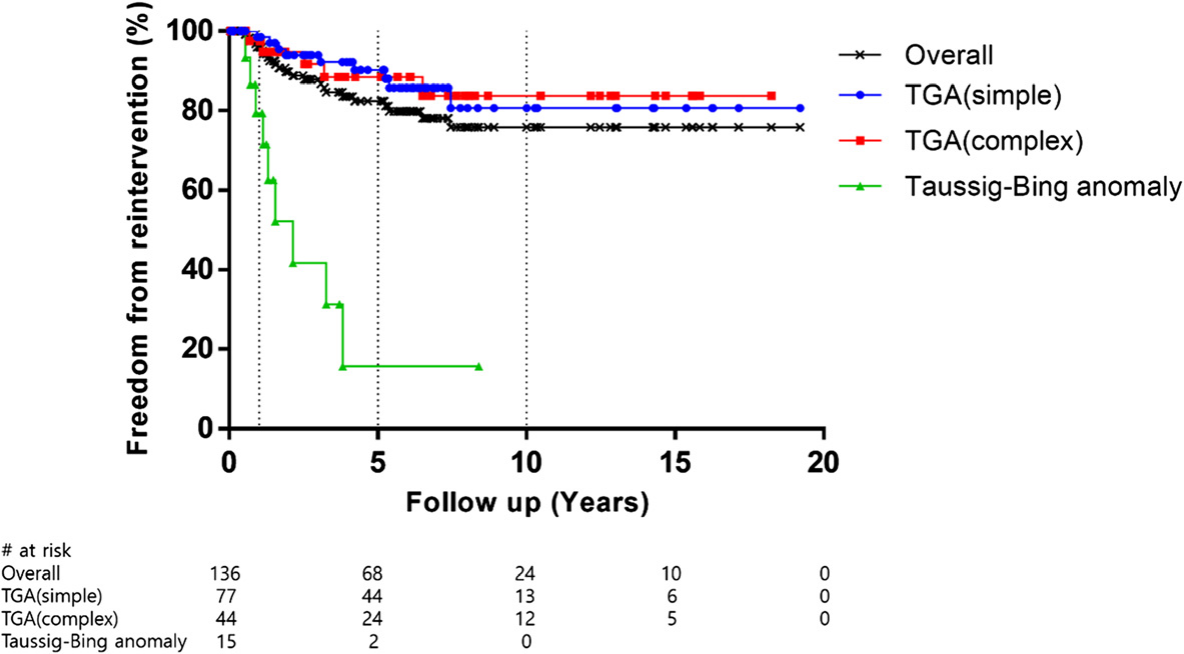
Freedom from reintervention for 136 survivors after arterial switch operation. Freedom from reintervention rate was lower (*p* < 0.001) in the Taussig–Bing anomaly group than those in the others. Simple transposition of great arteries (TGA) versus complex TGA, *p* = 0.952; simple TGA versus Taussig–Bing anomaly (TBA), *p* < 0.001; complex TGA versus TBA, *p* < 0.001

As shown in Fig. 2, The overall freedom from reintervention rates were 95.9 ± 1.8 % at 1 year, 82.5 ± 3.7 % at 5 years, and 75.8 ± 4.7 % at 10 years, respectively. One- and 5-year freedom from reintervention rates were significantly worse (*p* < 0.001) in the TBA group than those in the other groups: 79.4 ± 10.6 % at 1 year and 15.6 ± 13.2 % at 5 years in the TBA group, 98.5 ± 1.5 % at 1 year and 90.2 ± 3.8 % at 5 years in the simple TGA group, and 97.5 ± 2.5 % at 1 year and 88.5 ± 5.5 % at 5 years in the complex TGA group. There was no significant difference in the incidence of reintervention between the simple and complex TGA groups (*p* = 0.952). The median interval between ASO and the first reintervention was 22.8 (6.4–89.2) months and median interval between ASO and the first reoperation was 25.6 (6.4–89.2) months.

The most common primary indication for reoperation was pulmonary tract pathology (*n* = 12) including pulmonary valve stenosis and RVOT stenosis in 6 patients, supravalvar pulmonary artery stenosis in 5, and pulmonary artery aneurysmal dilatation in 1. The second most common primary indication was systemic tract pathology (*n* = 5) including LVOT stenosis in 2 patients, aortic valve regurgitation in 1, aortic root dilatation in 1, and retroesophageal aortic arch in 1. Two patients underwent coronary artery ostial angioplasty because of coronary ostial stenosis at 16.3 and 62.9 months, respectively, after the ASO. We performed a patch enlargement of coronary ostium using a native pulmonary artery patch. Both patients remained asymptomatic without a stenosis until recently. The first reoperation procedures are detailed in Table 3. The follow-up periods after the reoperation were 9 years and 6 months, respectively. Thirteen of the 19 patients (68 %) who underwent reoperation required concomitant procedures for minor coexisting lesions and the most frequently performed concomitant procedure was pulmonary artery angioplasty (*n* = 10). Including the primary procedures, 16 patients (84.2 %) underwent pulmonary artery angioplasty during their first reoperation. The interval between the ASO and the first reoperation was not significantly different between groups according to the location of primary lesions (*p* = 0.584). Details of the first reoperation procedures are presented in Table 2. A multivariate analysis showed that diagnosis of TBA (hazard ratio, 7.09; *p* < 0.001) and side-by-side great artery relationship (hazard ratio, 7.98; *p* = 0.001) were independent risk factors for reoperation.

**Table 3:** Procedures performed during the first reoperation

	Primary procedure	Concomitant procedure	Median interval between ASO and first reoperation, months (range) *p* = 0.584
Pulmonary tract lesion in 12 patients	RVOT widening with pulmonary valvotomy, 4	PA angioplasty, 2 TV repair, 1 Residual VSD closure, 1	31.9 (6.4–89.2)
	Transannular RVOT patch, 2	PA angioplasty, 1 TV repair, 1	
	PA angioplasty, 6	PV repair, 1	
Systemic tract lesion in 5 patients	LVOT widening, 2	PA angioplasty, 2 AV repair, 1	20.0 (8.5–39.0)
	AV repair, 1	PA angioplasty, 1 Aortoplasty, 1	
	Aortoplasty, 1	PA angioplasty, 1	
	Distal aortic arch translocation, 1	PA angioplasty, 1	
Coronary artery lesion in 2 patients	Coronary ostial angioplasty, 2	PA angioplasty, 2	39.6 (16.3–62.9)

ASO, arterial switch operation; RVOT, right ventricular outflow tract; PA, pulmonary artery; TV, tricuspid valve; VSD, ventricular septal defect; LVOT, left ventricular outflow tract; AV, aortic valve

#### Neoaortic regurgitation

Among 136 early survivors, echocardiographic follow-up was completed in 129 patients (94.9 %). The median echocardiographic follow-up duration was 5.4 (0.02–18.8) years. Two patients underwent a reoperation for neoaortic valve regurgitation. One patient underwent a repair at 8.5 months after the ASO, primarily for severe regurgitation, and the other underwent repair for mild-to-moderate regurgitation concomitantly with another primary procedure. At the most recent echocardiographic follow-up, 36 of 129 (27.9 %) had at least mild neoaortic regurgitation; of these, the regurgitation was mild in 28 patients (77.8 %), mild to moderate in 3 (8.3 %), moderate in 4 (11.1%), and severe in 1 (2.8 %). The neoaortic regurgitation at the last follow-up is described in Table 4. The incidence of significant (at least moderate) neoaortic regurgitation was 3.9 %. The patient who underwent aortic valve repair for severe regurgitation remained with severe regurgitation at last follow-up and died before their second reoperation. Freedom from significant (at least moderate) neoaortic regurgitation was 97.0 ± 1.7 % at 5 years, 95.2 ± 2.5 % at 10 years, and 95.2 ± 2.5 % at 15 years. In multivariate analysis, diagnosis of TBA was a risk factor for at least moderate neoaortic regurgitation (hazard ratio, 8.26; *p* = 0.035). We did not find a statistically significant difference in the development of significant (at least moderate) AR between open and closed coronary transfer techniques. However, despite of longer follow-up period, only 2 of 38 patients who underwent open coronary transfer developed significant AR. In terms of neo-AR, the older open technique is not inferior to closed technique. But the closed coronary transfer technique is easy to perform, convenient to confirm bleeding from anastomosis site, and reproducible for unexperienced surgeons. We need more longer-term follow-up data to compare these techniques, because the number of events was small.

**Table 4:** Neoaortic regurgitation at the last follow-up

n (%)	Simple TGA	Complex TGA	Taussig–Bing anomaly	Total	*p*
	*n* = 72	*n* = 43	*n* = 14	*n* = 129^a^	
Mild	17 (23.6)	9 (20.9)	2 (14.3)	28 (21.7)	0.822
Mild to moderate	1 (1.4)	1 (2.3)	1 (7.1)	3 (2.3)	0.329
Moderate	2 (2.8)	1 (2.3)	1 (7.1)	4 (3.1)	0.557
Severe	0	0	1 (7.1)	1 (0.8)	0.109
Total	20 (27.8)	11 (25.6)	5 (35.7)	36 (27.9)	0.746

TGA, transposition of great arteries

^a^Among the 136 hospital survivors, echocardiographic follow-up was completed in 129 patients

#### Event-free survival rate

**Fig. 3:**
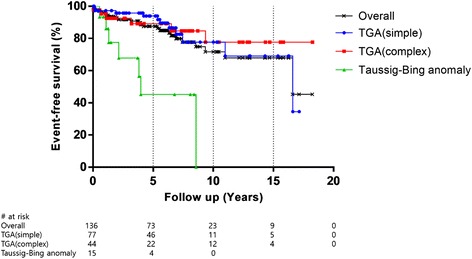
Kaplan–Meier estimates of event-free survival for 136 survivors after the arterial switch operation. Events included late death, reoperation and at least moderate neoaortic regurgitation. Taussig–Bing anomaly (TBA) group showed a lower (*p* < 0.001) event-free survival than the others. Simple transposition of great arteries (TGA) versus complex TGA, *p* = 0.763; simple TGA versus TBA, *p* < 0.001; complex TGA versus TBA, *p* = 0.001

The events in this study were defined as late death, reoperation, and at least moderate neoaortic regurgitation. Event-free survival rate was 87.5 ± 3.2 % at 5 years, 71.8 ± 6.0 % at 10 years, and 68.0 ± 6.8 % at 15 years (Fig. 3). Event-free survival rate was similar (p = 0.763) for TGA groups, and the TBA group showed a significantly lower (*p* < 0.001) event-free survival than the TGA groups: 45.5 ± 15.9 % at 5 years in the TBA group, 93.9 ± 3.0 % at 5 years in the simple TGA group, and 89.2 ± 5.2 % at 5 years in the complex TGA group. Multivariate analysis showed significantly poorer event-free survival in patients with TBA (hazard ratio, 5.548; *p* < 0.001).

## Discussion

The low early mortality (2.2 %) after ASOs found in this study is consistent with the findings of other recent studies of large-volume centers [5, 6, 9–12]. Karamlou et al. [9] showed the small-volume center or surgeon performing the ASO can be associated with high early mortality and morbidity, and recently small-to-medium centers [13–15] still reported high early mortality. Karamlou et al. demonstrated that the surgeon volume more influence early outcome than the center volume [9]. We have three surgeons and each surgeons performed 2.32 cases/year. We are able to confirm that our center and surgeon volumes are low.

Popov et al. reported improvement in early outcomes, which were poorer during the initial period [16]. Among our 3 in-hospital deaths, cardiac deaths only occurred in 2 patients within the first 3 years. Besides the patient who died of sepsis, there was no cardiac death and extracorporeal membrane oxygenation support has not been necessary since 1998. The present study shows that ASO can be performed safely, even in a small center. As expected from other reports [12, 17], multivariate analysis revealed that low body weight (<3 kg) was the only significant risk factor for early death.

We could not determine the relationship between coronary anomalies and early mortality. Although coronary anomalies have usually been considered a strong risk factor, recently some reports suggested that an unusual coronary anatomy is not a risk factor [12, 15, 16]. Because there have been many advances in surgical techniques in the past 2 decades, especially in those for coronary transfer, we believe that the technical advances and current experience may offset the impact of coronary anomalies. In more than two-thirds of patients who underwent ASO more recently, coronary artery buttons were transferred after completion of neoaortic anastomosis, which made it easy to determine the optimal site for coronary transfer. We could not determine the significance of these techniques because of time limitations and the small sample size. A larger sample size will be necessary for further studies to conclude a causal association. However, a coronary anomaly can make the procedure more difficult to perform. Postoperative myocardial ischemia is the most lethal complication. Myocardial ischemia is the most common cause of early death. Some recent studies reported that coronary anomaly remains a risk factor for early mortality [5, 12, 18, 19]. Although we should take note of coronary anatomy, our results and those of other recent studies imply that the continued development of surgical techniques for preventing coronary stenosis may improve survival rates.

By contrast with earlier reports [19, 20] that complex TGA results in a higher mortality rate than simple TGA, we did not find any association between other anomalies and mortality. We performed aortic arch repair in 15 (10.8 %) patients simultaneously with the ASO and found concomitant aortic arch repair is not a risk factor. One-stage repair for the TGA associated with interrupted aortic arch or coarctation of aorta is reasonable.

Among 136 hospital survivors, there were 3 (2.2 %) late deaths, which showed a long-term survival rate of 97.6 % at 15 years, which was comparable to that found in other studies [11, 12]. Two patients died within 2 months of hospital discharge. Considering the abruptness of the deaths in those patients with no problems during postoperative hospital course, the cause of their death might have been coronary ischemia. Reoperation for significant coronary artery stenosis was performed in 2 patients. Those patients had no symptoms or signs of coronary ischemia. Coronary stenosis was detected by routine coronary artery angiography. Previous studies showed that most patients have clinically silent coronary lesions, which were not evident on electrocardiography or echocardiography [21, 22]. Careful and prolonged regular follow-up using coronary angiography appears mandatory for optimal patient survival.

Although the ASO has been the treatment of choice for the TGA over the past 3 decades because of its excellent survival rate, a relatively high reintervention rate remains a problem. Our late reintervention rate and freedom from reintervention rate were similar to other centers [11–13, 15], and the reoperation rate was also comparable to others [20, 23]. The reoperation rates between simple and complex TGA were similar, but higher for TBA.

We found that the most common indications for reoperation were pulmonary tract lesions, especially supravalvar pulmonary stenosis, as has also been reported by others [11–13, 16, 20, 23]. Because there are many anatomical and technical factors influencing pulmonary lesions, their incidence varies considerably [12, 19, 20, 23–25]. We usually reconstructed the neopulmonary root using a glutaraldehyde-fixed pantaloon autologous pericardial patch, and 12 (8.8 %) patients underwent pulmonary tract reoperation mainly because of pulmonary stenosis in 11 and pulmonary artery aneurysm in 1. Including 3 patients who underwent balloon angioplasty for pulmonary stenosis, the reintervention rate was 11 %. Pulmonary reoperations were performed in the earlier postoperative period with a median interval of 31.9 months. One half of patients had RVOT obstruction with significant pulmonary valvar stenosis and underwent RVOT widening. Two patients underwent RVOT widening with transannular patch reconstruction, which has the potential to require reoperation. One of these patients underwent a third reoperation for RVOT obstruction. It is important that the initial ASO be performed carefully to reduce the possibility of pulmonary stenosis by considering various factors. We completely mobilized the great arteries and extensively mobilized the pulmonary artery to the hilum to create a tension-free anastomosis. Great arteries are divided at the lower level of ascending aorta and at the higher level of the pulmonary artery to avoid forming a long neoaorta, and we used a pantaloon autologous pericardial patch as mentioned above. One recent study showed excellent midterm results with minimal supravalvar pulmonary stenosis [26].

We found that the most common indication for late reoperation for systemic tract lesions was LVOT obstruction, not neoaortic regurgitation. LVOT obstruction after the ASO is a rare complication with an incidence of 0.59 % [27]. LVOT obstruction occurred frequently in patients with TBA or preoperative LVOT anomalies or a significant pressure gradient [27]. We found that 2 patients underwent reoperation mainly because of LVOT obstruction. One had TBA, which had a malaligned conal septum, and the other posterior TGA with VSD had subaortic accessory chordae, which were resected during ASO. We note that patients with preoperative conditions (such as a significant pressure gradient, TBA, abnormal chordae insertion, or muscular hypertrophy), which can be a risk of LVOT obstruction should have them properly managed simultaneously during the first operation.

Like other studies that reported excellent neoaortic valve function [28, 29], the incidence of moderate or severe neoaortic regurgitation was 3.9 % and aortic valve repair was necessary in 1 (0.7 %) of these patients. However, the development neoaortic regurgitation increases with time [28]. The median echocardiographic follow-up duration of 5.4 years in our study was shorter than in other larger series [11, 28, 30, 31]. Therefore, further long-term follow-up data are necessary to clarify the development of neoaortic regurgitation.

This study has several limitations, including its retrospective nature, limited sample size, and single center design. The small sample size and small number of events can influence the significance of our findings. Our follow-up period was relatively short for definitive evaluation of the definite long-term effect of the ASO for neoaortic valve function.

## Conclusions

We showed that the ASO can be performed with good early results and favorable long-term outcomes even in a small-volume center. Adequate and precise coronary artery transfer may improve survival rates regardless of coronary anomalies. The risk of late reintervention is low and TBA was identified as a risk factor for late reintervention. Although the incidence of development of significant neoaortic regurgitation is low in this study, a longer-term study will be necessary to evaluate the actual effect of ASO on neoaortic valve function. Close life-long surveillance is mandatory to detect structural or hemodynamic changes and to assess the true results of ASO.
